# New Diagnostics for Yaws

**DOI:** 10.4269/ajtmh.16-0639

**Published:** 2017-01-11

**Authors:** Christian Kositz, Robert Butcher, Michael Marks

**Affiliations:** 1 Clinical Research Department, Faculty of Infectious and Tropical Diseases, London School of Hygiene and Tropical Medicine, London, United Kingdom.; 2 The Hospital for Tropical Diseases, London, United Kingdom.

**Figure 1.**
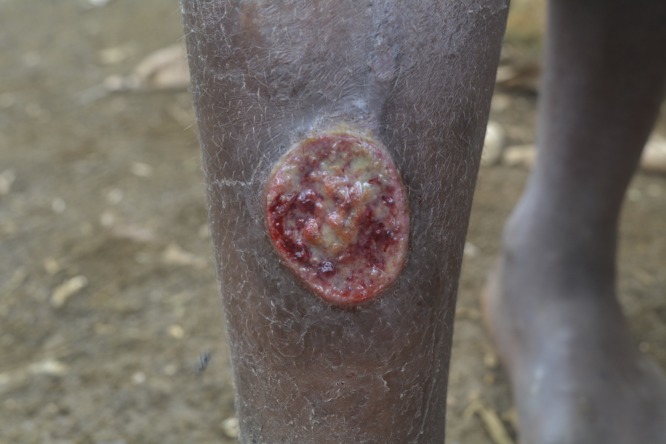
Classical primary ulcer of yaws.

**Figure 2.**
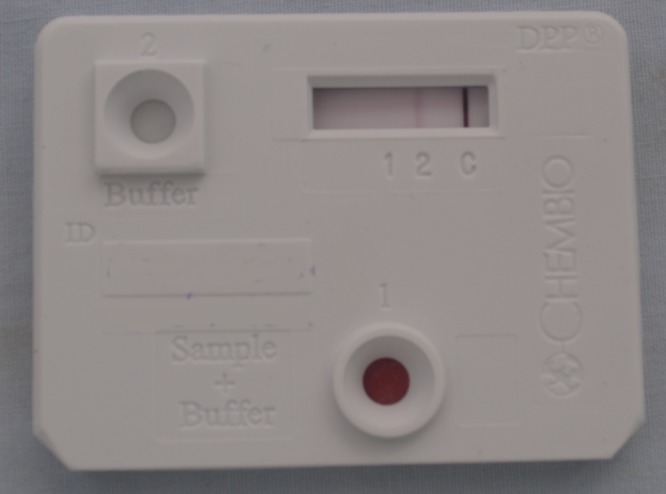
Combined treponemal and nontreponemal rapid diagnostic test for yaws.

**Figure 3.**
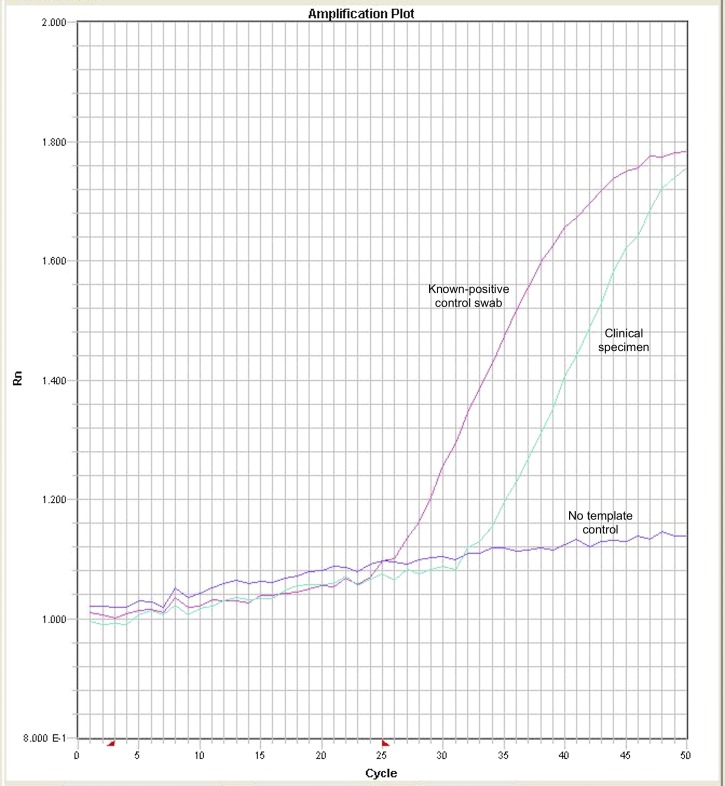
Quantitative polymerase chain reaction for yaws.

Yaws, caused by *Treponema pallidum* subsp. *pertenue*, is an important public health problem in many tropical countries.^1^ Like syphilis, the disease manifests in three stages; however, unlike syphilis, its route of transmission is non-genital skin-to-skin contact and not by sexual intercourse. Primary yaws manifests as either a papilloma or a chronic ulcer. Typically, ulcers are painless, with a raised edge and friable base (Figure 1

). In secondary yaws, skin manifestations, involvement of the bones and joints including periostitis have been reported. Tertiary yaws develops in a minority of patients causing destructive lesions of the skin and soft tissues. Interest in yaws has been revived by the finding that azithromycin is a highly effective treatment of both primary and secondary yaws.^2^ Clinical diagnosis alone of primary yaws is unreliable, but a point-of-care test has been shown to be of value.^3^ This test provides a result analogous to a *T. pallidum* particle agglutination assay (Figure 2

, line 1) and a rapid plasma reagin (RPR) assay (Figure 2, line 2). In early infection, only the RPR may be positive. Diagnosis has been further complicated by the discovery that *Haemophilus ducreyi* may cause clinically similar ulcers.^4^ New polymerase chain reaction (PCR) assays have been developed for yaws.^5^ DNA suitable for can be extracted directly from swabs collected into dry tubes without the need for transport medium. Figure 3

demonstrates real-time PCR amplification curves of positive and negative controls and a clinical swab from a yaws lesion containing *T. pallidum pertenue* DNA. Both serological and molecular tests have a major role to play in the World Health Organization yaws eradication campaign.
